# Vulnerability, loss, and coping experiences of health care workers and first responders during the covid-19 pandemic: a qualitative study

**DOI:** 10.1080/17482631.2022.2066254

**Published:** 2022-04-20

**Authors:** Suzanne C. Smeltzer, Linda Carman Copel, Patricia K. Bradley, Linda Tina Maldonado, Christine D Byrne, Jennifer Dean Durning, Donna Sullivan Havens, Heather Brom, Janell L. Mensinger, Jennifer Yost

**Affiliations:** a Nursing for Vulnerable Populations, M. Louise Fitzpatrick College of Nursing, Villanova University, Villanova, PA, USA; b M. Louise Fitzpatrick College of Nursing, Villanova University, Villanova, PA, USA; c Department of Clinical and School Psychology, Nova Southeastern University, Fort Lauderdale, FL, USA

**Keywords:** stress, loss, vulnerability, coping, work environment, pandemic

## Abstract

INTRODUCTION. The ongoing COVID-19 pandemic substantially affects health care workers from multiple disciplines, including nurses, physicians, therapists, and first responders. The aims of this study were to 1) explore and describe the experiences of health care workers and first responders working with individuals with COVID-19 infection, and 2) identify the support and strategies that were helpful during their experience.METHODS. A qualitative descriptive study was conducted via online video interviews of 29 health care workers and first responders who agreed to be contacted for an interview. Thematic analysis resulted in three themes and corresponding subthemes.RESULTS. The three overriding themes were 1) experiencing vulnerability, 2) suffering loss and grief, and 3) coping with vulnerability. A sense of vulnerability and high levels of stress were described and affected participants during their professional work as health care workers and first responders as well as their roles in their homes and communities.DISCUSSION AND CONCLUSION. The findings indicate the need for effective measures to assist health care workers and first responders to minimize the negative consequences of persistent and severe stress and vulnerability as they care for individuals with COVID-19 and their families.

## Introduction

COVID-19 is the first global pandemic in more than a century. It has resulted in over 423 million cases globally and over 5.9 million deaths as of 20 February 2022 (World Health Organization [WHO], 2022). In the U.S. alone, there have been more than 78 million cases and over 935,000 deaths (Centers for Disease Control and Prevention [CDC], 2022). These estimates are likely lower than actual cases because of asymptomatic cases, lack of optimal testing, COVID-related deaths in which COVID was not diagnosed, or deaths prior to individuals seeking health care. Although many individuals who developed COVID-19 experienced relatively minor symptoms and recovered without serious sequelae, others required prolonged time in intensive care units (ICU). This surge in demand for intensive care services resulted in many hospitals creating temporary COVID-19 units to accommodate the large numbers of affected persons who required mechanical ventilation for treatment of severe acute respiratory syndrome and respiratory failure. Survivors of severe cases of COVID-19 have reported significant residual symptoms, including pulmonary issues, peripheral and central nervous system symptoms related to immunologic responses, and others. These include serious sequelae because of the cytokine storm, such as cerebral oedema and multisystem organ failure (Fiani, Covarrubias, Desai, Sekhon & Jarrah, 2020). Some patients required lung transplants to survive (Bharat et al., 2020).

The number of health care workers infected with SARS-CoV-2 around the world is unknown, but the estimated number of health care workers who have died from COVID-19 between January 2020 and May 2021, is 80,000 to 180,000, with a population-based estimate of 115,500 deaths, although the actual number is thought to be considerably higher (WHO, 2021). More than 929,000 diagnosed cases of COVID-19 have been reported among health care workers in the U.S. with more than 3,600 deaths among health care providers as of 19 February 2022 (CDC, 2022). Health care workers (e.g., nurses, physicians, therapists), other personnel (e.g., first responders, such as emergency medical technicians, firefighters, police), and those who support patients and health care workers (e.g., environmental services, housekeeping, dietary services) are at greater risk for COVID-19 because of their proximity to affected patients. In addition to their increased risk of COVID-19, health care personnel, first responders, and those in supportive roles are at risk for the post-infection sequelae and for the effects of severe stress associated with the COVID-19 pandemic.

Infection and even death due to SARS-CoV-2 underestimate the impact of the COVID-19 pandemic on health care workers and first responders. In a recent scoping review of 37 quantitative and qualitative studies on the impact of COVID-19 on health care workers’ wellness, Shreffler, Petrey and Huecker (2020) identified consistent reports of major stress, anxiety, and depressive symptoms in health care workers in multiple countries due to COVID-19. In a second scoping review of psychosocial risks to health workers during the pandemic, Franklin and Gkiouleka (2021) analysed 220 articles from multiple countries with a variety of health care workers. Those articles that included primary data identified the major psychosocial burden of COVID-19 on health care workers who were nurses and women, resulting in post-traumatic stress symptoms, burnout, fatigue, and physical and emotional exhaustion. Franklin and Gkiouleka pointed out that the pandemic intensified pre-existing psychosocial risk factors in health care workers.

Arcadi et al.’s (2021) phenomenological study of experiences of 20 hospital-based nurses in Italy during the pandemic revealed uncertainty and fear of the unknown to the point of disruption and disorientation. The nurses reported altered perceptions of time and space, blurring of their roles and relationships, and alterations in their care of the sick and dying to prevent transmission of the virus. Although they reported strong support from other professionals, abandonment by administration was also described. Arcadi et al. identified recognition of the importance and value of nurses as a positive outcome of the pandemic and called for psychological support and training of nurses to increase their readiness for similar extreme situations in the future.

Bennet et al. (2020) analysed anonymous Twitter postings of 54 front-line health care workers to explore their experiences while caring for patients with COVID-19 in the first wave of the pandemic in the United Kingdom. The postings from front-line health care workers, including nurses, physicians, physical therapists, radiographer, health care assistant and others, described experiences as both immensely rewarding and profoundly traumatic. They reported feeling inadequate and overwhelmed. They were affected mentally, emotionally, psychologically, and physically and described that such distress extended to their patients who survived SARS-CoV-2 infection reporting that patients experienced psychological trauma as well as the physical consequences of having COVID-19. Because of the consequences of caring for patients during the pandemic, Bennet et al. described health care workers as “second victims” of the COVID-19 pandemic.

Although these study findings were not available at the time the present study was designed and conducted, they provide a strong rationale to further examine the experiences of health care workers and first responders during the COVID-19 pandemic. The current study is relevant and important because the pandemic persists, its duration is uncertain, and it is unlikely to be the last pandemic. Further, those with high stress levels left unresolved may experience continuing psychological distress. The experiences of front-line workers health care workers and first responders are important to discern and to understand to guide the development of strategies to support these workers through pandemics such as COVID-19 and the aftermath of such events. Because of the events and risks encountered by individuals caring for those with COVID-19, it is essential to explore and describe the experiences of the workers who deliver direct patient care as well as those who provide non-clinical services for COVID-19 patients in hospital and community settings.

## Purpose of the study

This qualitative descriptive study is part of a larger study, the COVID-19 Study and Registry of Healthcare and Support Personnel (CHAMPS), conducted to investigate the effects of working in health care and related settings during the evolving pandemic on the long-term health of health care personnel, support personnel, and first responders working in health care facilities or in the community throughout the United States (Kaufmann et al., 2021; Mensinger et al., 2022). The purposes of this portion of the CHAMPS study were to: 1) explore and describe the experiences of individuals who work or worked with individuals with COVID-19 as health care professionals (e.g., nurses, physicians, therapists [respiratory, physical, and speech], etc.), first responders (emergency medical technicians, police, firefighters, ambulance drivers, etc.), and essential personnel in health care facilities (environmental services, dietary staff members, housekeeping, etc.), and 2) identify the supports and strategies that were helpful to them during the experience.

## Methods

Participants were recruited from the CHAMPS study, which enrolled health care workers and first responders who responded to an outreach email disseminated through a variety of organizations, including alumni societies, news media, professional and trade groups, social media, and other facilities that treated or interacted with patients with COVID-19. This initial CHAMPS cohort was enrolled in the study in the spring and summer of 2020.

At the time of recruitment for the qualitative component of the CHAMPS study (September 2020-January 2021), 457 (45.7%) of the initial 1,000 individuals who had participated in the larger CHAMPS survey in the spring and summer of 2020 had indicated that they were willing to be contacted for interviews. Multiple emails were sent to each of the first 305 (66.7%) of these 457 participants to determine their continued willingness to participate in interviews and to schedule interviews with them. Lack of response to these emails, scheduling conflicts related to work/family responsibilities, and individuals’ decisions not to participate in interviews because they did not want to revisit or talk about their experiences resulted in interviews of 29 (6.3% of those who initially indicated a willingness to be interviewed and 9.5% of those the researchers attempted to contact for interviews).

Participants in CHAMPS who indicated a willingness to be contacted for a possible interview and provided their email addresses were contacted by email and provided a concise description of the interview portion of the study. If they responded by email that they were still willing to be interviewed, we sent them a consent form and a virtual meeting was scheduled for the interviews. Each interview began asking participants if they had any concerns or questions about the study and they were asked to agree verbally to participate in the study. Using a semi-structured interview guide, two researchers (S.C.S., L.C.C.) conducted and recorded interviews via Zoom between September and December 2020. Interview questions asked participants about their work role and their experiences working with patients with COVID-19, what barriers they encountered, what coping strategies they used to deal with their experiences, and if they had anything else they wanted to share with the interviewers. The interviews, which ranged in duration from 40 to 60 minutes, were conducted and recorded by Zoom, resulting in verbatim transcriptions of the interviews. The transcriptions were validated for accuracy by a member of the research team (C.D.B) who listened to each recording while reading and correcting the transcription. All identifying information was deleted from the transcripts. Interviews were stopped when redundancy in responses was detected, which occurred with 26 participants; interviews were conducted with the remaining three participants who had interviews scheduled. The qualitative study as well as the parent CHAMPS study received Institutional Review Board (IRB) approval as an expedited study.

Thematic analysis (Braun & Clarke, 2006) was used to analyse the 29 transcripts. The analysis was conducted manually. Each of the validated transcripts was read by six members of the research team (S.C.S., L.C.C., P.K.B., L.M., C.D.B., J.D.D.) who identified meaningful statements in each transcript. The statements were organized with labels reflecting common statements made by the participants. These labels were discussed in several meetings by the six-member data analysis team to identify and agree on themes that reflected responses of the study participants.

The study’s trustworthiness and rigour were addressed by a collaborative approach to all phases of the study, prolonged researcher involvement with the data, inclusion of frontline workers as members of the research team, and reflection of the research team on data and findings throughout the process and in multiple team meetings. The research team elected not to use member checking because of the risk of retraumatizing participants as they recalled distressing experiences.

## Participants

The study participants were primarily White non-Hispanic, married, female nurses, along with two dieticians, a police officer, radiographer, and emergency medical technician (EMT). They were employed in inpatient facilities, outpatient facilities, and community-based agencies. Twenty-two were employed in the Northeast region of the U.S., with the remaining seven from other regions of the U.S. Table I provides additional demographic information on the participants.

**Table I.** Demographic characteristics (n = 29)

Characteristic	N		(%)
Gender			
Female	26		(89.7)
Male	3		(10.3)
Race/Ethnicity			
White Non-Hispanic	28		(96.6)
Other	1		(3.4)
Age (years)			
20–30	5		(17.2)
31–40	10		(34.5)
41–50	6		(20.7)
51–60	6		(20.7)
> 60	2		(6.9)
Category of Professional Role			
Nurse	24		(82.8)
Dietician	2		(6.9)
Paramedic/EMT	1		(3.4)
Radiographer	1		(3.4)
Police Officer	1		(3.4)
Years in Job or Professional Role			
< 10 years	10		(34.5)
10–20	8		(27.6)
> 20	11		(37.9)
Work Site			
Emergency department	5		(17.2)
Intensive care unit (ICU)	8		(27.5)
Non-ICU COVID unit	4		(13.8)
Other: in-patient unit	3		(10.3)
Outpatient/non-hospital setting	3		(10.3)
Outpatient/hospital setting			
Other*	6		(20.9)
Region			
Northeast	22		(75.9)
South	3		(10.3)
Midwest	2		(6.9)
West	2		(6.9)
Relationship Status			
Married/Domestic Partner	20		(69.0)
Divorced or separated	2		(6.9)
Single	7		(24.1)
Usual Living Arrangements			
Live with spouse/partner	12		(41.4)
Live with spouse/partner & children	9		(31.0)
Single parent with children	2		(6.9)
Live with roommate or roommates	2		(6.9)
Live alone	4		(13.8)

*Home care, correctional facility, long-term care facility, police department, community emergency services, palliative care/outpatient hospice setting

## Results

Using Braun and Clarke’s (2006) thematic analysis approach, three themes and corresponding subthemes were identified and are presented in Table II along with illustrative examples from the interviews. Theme one: Experiencing Vulnerability consisted of three subthemes: 1) feeling emotionally vulnerable, 2) shouldering professional responsibilities, and 3) enduring community responses. Theme two: Suffering Loss and Grief had three subthemes: 1) losing control, 2) experiencing loss of lives, and 3) feelings of guilt and grief. Theme three: Coping with Vulnerability involved three subthemes: 1) gathering information and strategies, 2) seeking support, and 3) practicing self-care. Participants were primarily nurses, although the five non-nurse participants described experiences similar to those of the 24 participants who were nurses.

**Table II.** Examples of participants’ quotes for themes

THEME ONE—EXPERIENCING VULNERABILITY
**SUBTHEME 1: Feeling Emotionally Vulnerable**
In the beginning it was more physically draining because you were running on adrenaline, and you were doing what you had to do. And then … after days off is when it would hit you, or like when you were driving home is when it would hit you. But when you were there, you didn’t have time to really give a second thought to what you were doing because you … were so busy”.
**SUBTHEME 2: Shouldering Professional Responsibilities**
“ … our [nurses’] boundaries expanded exponentially.” “On one hand, they felt completely like they’re [nurses] running the whole show, and the responsibility is completely on them … ”
**SUBTHEME 3: Enduring Community Responses**
“You know, you have people who are going about their lives like nothing’s changed and you would like to do the same thing, but you know it’s wrong.”
**THEME TWO—SUFFERING LOSS AND GRIEF**
**SUBTHEME 1: Losing Control**
“I have never experienced anything like this, and I do not think I will again in my career.” “ … how disturbed we were by them playing ‘Here Comes the Sun’ every time somebody gets discharged … because it was happening while patients were dying in the ICUs.”
**SUBTHEME 2: Experiencing Loss of Lives**
“Some of the stories have been so sad like patients wanting to see their [loved ones] … they know they’re going to be intubated. And they’re like, begging for us to “hold up my phone. I want to see my wife’s picture. I want that to be the last thing I see.” “On a trauma level … just a lot of loss … a lot of young loss; and staff, I think we’re just completely broken at that point. So having the chaplain available. I mean, leading our morning huddle and a prayer. I mean it was just truly to just kind of get people together. In a way to say … ‘This is hard. Yeah, this is hard’ … ”
**SUBTHEME 3: Feelings of Guilt and Grief**
“ … I couldn’t save people at work, and I couldn’t save him [father] at home. It was really difficult.” “You’re not able to give them any real hope. Hopefully, you’re saying there’s been no change, like, in my experience, it’s very rare that someone improves once they’ve been intubated. So, but they’re all kind of clinging to the same questions. You know how much oxygen, are they getting?”
**THEME THREE—COPING WITH VULNERABILITY**
**SUBTHEME 1: Gathering Information and Strategies**
“I still, you know, I have this big ritual with a COVID patient I wrap up my work tablet. I sanitize … the whole … taking off and putting on PPE and sanitizing. And by the way, not everyone has done that in a wind and rainstorm, but I have many times.”
**SUBTHEME 2: Seeking Support**
“All summer I was looking for other jobs because, like, I can’t do that again.” “I considered myself a pretty resilient ICU nurse. And so, for me to just be ready to throw in the towel is pretty significant.” “Just talking about it and certainly I have support, I have family and certainly I have friends who are willing to listen. But they know me, and they love me and so there’s that emotional component … but there are somethings I hold back.”
**SUBTHEME 3: Practicing Self-Care**
“So actually, right when I got sick, I would reach out to do therapy because I’m already … so anxious all the time and I was living alone, and everything was long ago … but it actually has been … pretty bad recently that I had to go to … a doctor and get … medicine and what not because it’s just not resolving.” “I’m very proud of what we were able to accomplish in a very short period of time, with no notice essentially. So, there is a sense of pride in our work, in my work, in my peers’ work and certainly a sense of camaraderie with the people that we went through this with that, you know, we were able to get through it.”

## Theme one: experiencing vulnerability

During the initial months of the pandemic, as uncertainty and chaos unfolded, so too did feelings of vulnerability. Participants described experiencing a myriad of emotions resulting from physical, professional, and social/community encounters. Lack of knowledge about what the SARS-CoV-2 virus was, how to identify and treat COVID-19 disease, and how to stop its transmission fuelled this sense of vulnerability. Experiencing vulnerability led to feeling unprepared and unsafe due to the lack of training and the lack of physical and emotional support. Being under-resourced in staff and supplies and alone in shouldering responsibilities to manage patient care increased participants’ feelings of personal risk and vulnerability.

### Subtheme 1: feeling emotionally vulnerable

The fluctuating levels of anxiety experienced by the participants manifested themselves differently. All participants described an overwhelming fear of exposure to COVID-19 that extended beyond self to family and patients. The police officer expressed the following concern: *“What if I get this? I’m not symptomatic and I can take it home to my family.”* A nurse manager stated, *“I was scared … there was a staff breakout of COVID … many health care providers became ill at work and were sent home.”* One experienced ICU nurse stated, “*I think a lot of people have this feeling of inevitability like we’re just going to get it. You can only do so much to avoid it, but I was afraid of bringing it to my sister and my (newborn) nephew.”* In the absence of routine testing early in the pandemic, some wondered about transmission to the patients and questioned, *“Did we give (the virus) to him? We really don’t know who had it first, staff or the patient”.* Coming to work was an emotional trigger described as,*“I was always anxious going to work because I didn’t know what I’d be walking into that day.”* This nurse added, *“We were physically and emotionally exhausted and had a difficult time just catching our breath.”* The radiographer participant stated, “*the pandemic has really turned my world upside down, for months I did not hug my children*.” This participant rated the daily stress level as very high.

An experienced ICU nurse described the early months of the pandemic this way: *“In the beginning it was just complete chaos. We didn’t know what to expect … it was both physically and emotionally draining ….”*

Another ICU nurse whose spouse is a first responder remarked that she was *“uneasy, thinking about possible exposure to COVID-19”*. In preparation for possible exposure, she cleared out a shed in her yard in case she or her spouse needed to be isolated after being “really exposed” to COVID-19 to prevent exposing family members.

Many of the participants addressed not sleeping well and having nightmares. Several spoke about their experiences as similar to symptoms associated with post-traumatic stress disorder (PTSD). One experienced ICU nurse who had very recently returned to work after maternity leave shared that a psychotherapist explained to her that she was exhibiting and describing signs of PTSD. She realized that her work mentally affected her, but she found it “a little jarring” to have someone recognize that she was experiencing PTSD. She indicated that there were many traumatic events that occurred “on every single shift.” Another nurse participant reported, *“I was getting so anxious that I had several panic attacks during the surge … It disrupted my whole life … and kept me nervous and scared and in survival mode for a long time.”* Another nurse stated, *“There’s a lot of flash backing that happens … that sometimes kind of takes my breath away;”*

One experienced ICU nurse working in a large regional community hospital described her response to her experience: *“There’s days I just cry. You don’t even know why you’re crying. You’re just crying because it’s a lot.”* Another experienced ICU/ED nurse responsible for setting up new COVID units and training other nurses to care for critically ill patients stated,

*I’m not a crier, but … driving home I started to cry … I just couldn’t stop. And I came in the door … I sat down and grabbed my laptop, and just started typing … I needed to write it out … once I just got it out of my system I felt better. And I wrote … about the stresses of throwing these units together.*

A dietician stated, *“This has kept me up nights.”* A nurse participant summed up her feelings this way: *“The hardest thing about COVID was provider apprehension and provider fatigue.”*

### Subtheme 2: shouldering professional responsibilities

In a number of institutions, staff were told by the hospital administration, *“The nurse is going to be the one in the (patient) room and nobody else; ‘just don’t go in’ and ‘just tell them (the nurses) what to do (from the door of the room).’”* Because nurses were the primary caregivers of hospitalized patients, and often the only personnel permitted in patients’ rooms during the height of the COVID-19 pandemic, they felt responsible for meeting all the needs of the patients in their care and reported that the obligations of nurses never stopped. One ICU nurse stated, *“They tried to heavily limit the number of people in the room, so they up-trained the nurses to basically do everything.”*

Several nurses expressed feeling overwhelmed with the heavy workload of patient care, the need to carry out the functions of other personnel, and “*being responsible to do it all,”* including serving food trays, emptying trash containers, basic housekeeping in patients’ rooms, adjusting ventilator settings, and even anointing dying patients. They took on these additional roles because dietary services, housekeeping, patient care assistants, and clergy were not considered essential personnel who needed to enter patients’ rooms. When social workers interacted with patients, this often required the assistance of nurses in the patients’ rooms to hold the phone or set up the device for patients to talk with social workers. An experienced ICU nurse stated, “*Nurses were expected to … to do everything. If there was a vent (ventilator) in the room and you were having problems with it, the respiratory therapist would stand at the door and kind of coach you on what to do.”*

A nurse manager described her introduction to the COVID-19 pandemic this way:

*I walked into work on Monday morning, and I was told, ‘You have just inherited an adult COVID unit. You have two hours to open it. It is going to be on the peds floor, and your staff, the high-risk OB nurses, and the pediatric nurses will be staffing it. Your job is to make them figure out how to do it.’ My unit had 34 beds and it was at full capacity within an hour and 12 minutes.*

This nurse manager also reported quickly running out of supplies and PPE and the effect on nursing staff: *“We ran out very quickly. We started repurposing. The (nurses) were terrified. I had two nurses just quit. You know, I am not doing this. I am not safe.”*

A clinical nurse educator/ICU nurse described the early efforts to establish and staff new units for patients with COVID-19: *“It was mayhem because it was a bunch of staff that were out of their element initially. And the units were not meant to be ICUs.”* At the beginning of the pandemic, she started teaching two-day ICU refresher courses “*because some of these nurses have been out of the ICU for 22 years or more*.”

Nurses reported that they and many of their peers were working in clinical areas and with high levels of patient acuity out of their comfort zone, which was exacerbated by inadequate training. One nurse stated, *“Some staff have been plucked out of their unit and kind of dropped in this alien planet.”* One nurse new to her hospital had just completed orientation to the paediatric intensive care unit. When the unit was converted to a COVID-19 unit, she was providing care for adult patients, stating that she was “*totally … thrown off guard*.” She further reported that she and other paediatric nurses had high levels of anxiety because they had little experience caring for adult patients.

The frustration shared by hospital workers early in the pandemic also evoked thoughts about hospital administration. Several participants expressed feelings of annoyance, irritation, and anger about situations in which peers and administrators *“did not rise to the challenge of COVID.”* Several participants spoke about the mixed messages from administration, with one nurse stating, *“doctors who shut down their offices, and others … who were able to work from home to protect themselves while we (nurses) were at risk.”*

A nurse who worked in the emergency room reported:

*When one of the administrators walked through the ED (Emergency Department), he had his own personal N-95 on that was all neoprene with the little vent filter … and I had to wear my surgical mask. I couldn’t help but shake my head and think I wasn’t worthy enough to wear one all the time, but you are.*

Participants in the hospital and in community-based settings (e.g., home care, correctional facility, police department) described being asked to take risks with no provisions for adequate protection. The major physical protection concern involved personal protective equipment (PPE). The participating radiographer stated, *“I was distressed when I found out that I had 10 known exposures without having PPE.”* Despite the overwhelming expectations for participants, they often worked without adequate PPE and other supplies and resources, including a lack of up-to-date knowledge about appropriate protocols and procedures to care for patients with COVID-19. Several participants indicated that their facilities stored PPE under lock and key. There was a lack of a sense of safety due to the mandated reuse of PPE. Many expressed feeling *“made to justify the need for proper masks.”* A nurse manager shared, *“Another disheartening event was when we were told not to wear or to conserve N95 masks and PPE.”* In general, the feeling expressed was that administration was *“asking too much of us but not giving us the resources.”* Several participants who worked in community settings (e.g., home care, correctional facility) reported that they were responsible for providing their own PPE and sanitizing solutions at the beginning of the pandemic; they relied on donations of these supplies from friends and family members. One ICU nurse indicted that masks that were available were often ill-fitting, which limited the protection the masks afforded.

### Subtheme 3: enduring community responses

Many study participants described interactions with community members that contributed to participants’ sense of vulnerability. Communities appeared to have strong reactions not only to COVID-19 but also to workers who provided care to patients with the virus. Community reactions noted by participants included deep-seated fears of contracting SARS-CoV-2, as well as concerns about being physically close to those working with COVID-19 patients. Several nurses expressed feeling shunned and discarded. One shared the discomfort of being excluded: *“A lot of people didn’t want to be around me because I’m a nurse, or … say I couldn’t come to things because they didn’t feel comfortable around me … So that was kind of upsetting.”* The radiographer shared how the community’s fear also affected the children: *“My neighbor across the street would not let her kids come near my kids because of my occupation.”*

Juxtaposed with the communities’ deep fear was also the initial celebration of nurses and other frontline workers as heroes. One nurse participant shared that the community, including firefighters, perceived health care workers as heroes deserving of praise: *“The hospital is right across … from this huge apartment complex and … they were all clapping out their windows. And the fire department would … cheer for you right when you would come off shift. And that was pretty epic*.” Yet others did not share the sense of being heroes, based on the negative outcomes in patient care they were experiencing: *“ … slap this label of ‘oh you’re a hero,” so it makes it okay’ … I don’t feel like a hero at all. I’m not really saving anyone … ”* Additionally, after the newness of the COVID-19 phenomena receded, many participants felt as though they had been forgotten. Another nurse described the change in how health care personnel were viewed: *“Initially we were heroes and now it feels more like we’re supplies that can be picked up and put down*.” Participants felt that people around them were going on with their lives while health care workers and first responders were not only fighting an invisible foe but were no longer recognized for their personal risks by those outside of the hospital space and in the community. One nurse described feeling that health care workers *“went from heroes to zeroes.”*

As the battle against COVID-19 persisted in hospital spaces, the participants described becoming increasingly aware of some community members’ wilfulness in not taking safety measures surrounding COVID-19. Participants shared their continuing frustration over experiencing the increasing cruelty of the disease as they worked while at the same time witnessing many in the community refusing to take COVID-19 safety precautions, such as wearing a mask in public and adhering to social distancing recommendations. As one participant noted:

*It’s just getting worse … it’s just the community … It’s just unbelievably frustrating … ‘I’m telling you my first-hand experience and you’re still choosing to just not listen, or you think it’s somehow not going to affect you.’ You know … it’s painful.*

Several nurses expressed wanting to invite members of the community to experience first-hand what they were going through in their work with patients with COVID-19. One stated, *“I really wish people could spend like 20 minutes seeing what occurs with COVID. I think they would get over themselves and be a little bit less selfish or less stupid.”*

One emergency department nurse indicated that her father dismisses the seriousness of COVID-19 infection and believes that it is just like the flu. She also described her responses to the lack of those in the community to follow recommendations to protect themselves and others:

*It’s really, really stressful … millions of people don’t think that this is real or think that we’re overblowing it … they refuse to wear masks and refuse to wash their hands … simple things. It’s super stressful and an insult to all of us who are working directly with these patients. We’re trying our best not to be overwhelmed at work and taking care of people who we’re pretty sure aren’t going to make it out of the hospital alive. That’s extremely frustrating … and it adds to the stress that we already have … people think that we’re lying about what’s going on … and that’s stressful.*

As participants looked beyond their local community, one nurse expressed a sentiment shared by many: *“I was angry on so many levels … the whole systemic issue of PPE and the way that that was handled … by the government. I’m still pretty angry at the regulatory agencies and the way … stuff has been politicized.”*

## Theme two: suffering loss and grief

The theme of suffering loss and grief centred on the array of losses experienced by study participants, especially the loss associated with the changes and transitions occurring daily in the workplace as the first wave of the pandemic occurred. Lost to the participants was the ability to depend on routines and usual care procedures, along with working with familiar peers on the units in the speciality in which they had experience and expertise. Feeling like a novice created a sense of guilt about not achieving the usual level of quality care. Lost too were the abilities to comfort patients and their families and to save lives.

### Subtheme 1: losing control

All study participants addressed their sense of loss on many levels. They expressed losing control of their time, their schedules, and how to protect themselves. The nurse participants also spoke about the loss of having educators, administrators, social workers, as well as the other support personnel they had working alongside them prior to the pandemic. One nurse who was not ICU trained explained, *“Everything, everything fell on nursing and then we didn’t really have a say even if we wanted to.”* Not being able to use their level of expertise conveyed another loss: *“The nurses I’m working with … have been doing this (caring for patients) for a minimum of ten, and most of them for 25 to 30 years … They’re experts in their field and … now they’re not … they’re taking away their (nurses’) control of everything.”*

All participants expressed how loss was experienced at home as well as in the workplace. At home, the loss of routines and the need to self-isolate and maintain distance from family members and friends created sadness. One of the dieticians explained, *“My work is affecting how stressed I am at home. Some days all I speak to my husband about is that I am going to lose my job because I speak up. Today I threw a fit at work to get management to hear me.”* The participants’ families were operating differently as the health care workers and first responders were working and away from home while their children were often home attending school virtually. A nurse participant stated, *“I had a loss of control of my family life”* and described *“a roller coaster of different emotions.”* The effect of the loss was viewed as ongoing: *“The loss of control, the loss of empowerment … it was just phenomenal … and I still think we’re dealing with the effects of it with our staff.”*

### Subtheme 2: experiencing loss of lives

The phrase *“unexpected loss”* and *“one loss after another”* were phrases used by almost all participants to elucidate the *“unbelievable losses that were occurring.”* The most profound losses the participants described were the “*number of deaths experienced,”* the witnessing of disturbing, sudden or prolonged deaths of patients, and “*the suffering of patients dying without the presence of family members and the significant people in their lives.”* They spoke of “*doing everything we could to stop death (but) there were deaths every day, just one after another.”* A nurse from the emergency department stated: *“Patients in the ED would be talking to us … answering questions … They would leave not going to the ICU, but to the morgue. They were coming in too sick and declining too quickly for us to save them.”* Others who witnessed the deaths of COVID-19 patients shared comments similar to this description:

*COVID deaths were the worst deaths I have ever seen … the air hunger, the drowning and the look in their eyes was awful. The look of someone who cannot breathe. I had seen that look before, but not like this. It was sheer terror.*

Despite the high number and disturbing nature of the many deaths that participants experienced, inadequate efforts were made by administration to provide support to health care workers. One very experienced ICU nurse stated, “*The administration totally dropped the ball on that.”* She also stated that although there was an employee assistance programmes (EAP) in her hospital, staff were too overwhelmed to take advantage of the program. When she called the human resources department to locate the EAP, she left a message but never received a call back. Although support may have been available to staff, she indicted that none of the staff knew about its existence.

### Subtheme 3: feeling guilt and grief

Several participants expressed a sense of guilt due to not knowing whether the measures they had taken for patients reflected the correct protocol, their inability to provide the quality of care they usually gave, and the inability to save lives. One nurse participant explained:

*I wondered if a patient would have done better if we had done more … but then we didn’t know things and we didn’t want to expose anybody else to the virus. We were discouraged because we felt we had to save patients, but we often couldn’t.*

An experienced ICU nurse/clinical nurse educator indicated that newly trained nurses had caregiver guilt, wondering if patients would have had better outcomes “*if they had a real ICU nurse*,” rather than one newly trained for the COVID-19 pandemic. Participants spoke of how going from expert with years of experience to a novice in the management and treatment of COVID-19 led to feelings of guilt. One participant stated, “*I think that’s where a large level of guilt and stress came for us because we’re supposed to be … the experts but … we can’t be the experts … we had to learn as the situations were happening.”* In contrast, another nurse participant indicated feeling grateful yet guilty about working in a protected environment because of adequate PPE in the COVID unit and the fact that *“on the unit you know that all patients had COVID, whereas in other areas of the hospital or out in public, you don’t know who is infected and who is not.”*

An ICU nurse commented on the sense of futility in providing care, stating, *“And it just feels sort of futile … we’re used to making a difference and at least trying to or having a hope of recovery … Not many of them do well and get better.”* The sadness, grief, and guilt were summed up by one nurse participant who was working without adequate staff or supplies, including PPE, and no negative pressure rooms: *“I just hope families know that I wanted to do all those things more than anything.”*

## Theme three: coping with vulnerability

The theme of coping with vulnerability involved both active and avoidance coping strategies. Participants actively gathered information and approaches to manage COVID-19, sought emotional support, and developed self-care strategies to manage their feelings of vulnerability. Whereas many participants described their strategies as decreasing their sense of vulnerability, they also spoke of ongoing struggles. In addition, some positive outcomes were identified, such as bonding and developing resilience.

### Subtheme 1: gathering information and strategies

Participants reported managing their concerns by reaching out to others for support and information. Participants discussed seeking out additional information to allay their anxieties and to obtain the latest information about the virus. A dietician stated, “*I was going to the CDC website myself to get guidance, to get the latest updates … I thought the very least (administration) could be doing was basic information on CDC guidelines (for) the directions we were given.”* One nurse participant reflected similarly, *“I studied a lot at home. I was just constantly trying to look up how to best take care of these patients.”* Another dietician discussed the effects of gathering information and collaboration: “*Even the clinical things … We were investigating (cases) on our own … and finding resources and sharing information … I thought that was a good thing that came out of this, the collaboration that happened early on.”*

All participants spoke of developing decontamination rituals before entering their homes. As a nurse participant described:

*I would take off my shoes outside and put them in a plastic bag … I would strip down in the laundry room and put everything in the washer and put it on the sanitizing cycle … and go upstairs and take a shower and I wouldn’t do anything else. I had a ritual.*

Another nurse stated, *“Just coming home was exhausting. You have done your 12 or 13 hours, and now you come home, and it takes another 45 minutes or so to get everything off and clean.”*

Other participants described not going home at all and staying elsewhere to protect their families, whether from transmission of COVID or from seeing the emotional and physical toll that the work was having on the workers. One nurse participant expressed: *“Often we were staying at hotels after doing a couple of 12-hour shifts in a row, as we were too tired emotionally or physically, to go home, or family didn’t want us to come home.”*

### Subtheme 2: seeking support

Because of staffing changes, participants felt that supportive co-workers were often unavailable. Although other colleagues were identified as supportive, which helped participants to cope with their stress, some avoided their co-workers outside of work to avoid “*rehashing stressful events*” and to avoid unhealthy ways of coping, such as increased alcohol intake.

Several nurse participants indicated that they had reconnected with previous therapists to deal with emotional responses to the stressful work environment. One nurse new to her health care institution indicated that not knowing co-workers well resulted in inability to trust co-workers in a support group. Others indicated that the availability of support strategies (e.g., counselling sessions, support groups) was not well disseminated, so staff were unaware of them and consequently did not take advantage of them. Some participants, however, did find support from their co-workers, including a nurse participant who stated,

*I’m really grateful those nurses that were working with me always looked out for me. I mean, they would have seven patients of their own and they were always checking on me. I felt really supported … Granted, I was alone most of the time, (but) everyone would stop what they were doing and help me when I needed (it).*

Several ICU nurses indicated that they tried to suppress their emotions because they felt unable to change the stressful situation and work environment. One approach used was to dissociate from the situation: “*I really did have to shut out a lot of my emotions about that because I knew that I couldn’t fix it … so you just sort of block it out*.” In response to their experiences, several participants were rethinking their career choices and reconsidering their future plans. One nurse stated, *“I just don’t think I want to be … in a hospital like this forever … understaffed … underpaid and overworked. I … reached out to restart school … so I can do … something else not at the bedside.”* Another nurse stated, *“I definitely think it’s made me re-think my career … I just feel like nurses are … super under-appreciated.”*

Another nurse summed up the experience and its effect on career choices:

*It’s making nurses that I know not want to stay at the bedside … They’re either going to get out sooner and start CRNA school sooner so that they’re not at the bedside or switching to something very different or taking a break from nursing and … teaching their kids at home.*

One participant, an emergency medical technician (EMT), identified the potential effect of the pandemic on the number of individuals training to become EMTs and the disruption of EMT training due to COVID-19. He indicated that several EMTs resigned because of the risks associated with exposure to patients with COVID-19.

### Subtheme 3: practicing self-care

Participants used a variety of self-care strategies to cope with the extremely high level of stress they experienced in their roles as health care workers and first responders caring for large numbers of critically ill and often dying patients. Some of the participants’ usual self-care strategies, however, were hampered by the limitations in place because of the pandemic. Several participants indicated that they used self-talk or journaling to help manage their stress. One nurse used journaling: *“Especially when I’m like panicking … I write down stuff to discuss with her (therapist) kind of honestly, to write it down and talk myself through it”.* Several others, including a clinical nurse educator, indicated that *“just talking about it”* was helpful, but one stated, *“There’s some things … I hold back because I don’t want them [family] to be sad. I don’t want them to carry it with them.”* Several nurse participants indicated that they tried to *“just let things go that I have no control over and try to focus where I can make a difference.”* One primary care nurse practitioner whose practice included patients with COVID-19 infection and complex medical problems discussed her fears and those of other workers:

*I had two coworkers who were scared to come to work … initially I was too. And then I thought, I can’t live like this. I am the one who says ‘go with the science.’ I have to be effective … I can’t be anxious all the time.*

She also reported that she takes steps to avoid being frustrated, but “*I carry on to my family …. that’s my coping mechanism*.”

A few participants mentioned prayer as a coping strategy. Others described restarting exercise programmes and taking up previous hobbies such as photography, yoga, and “*watching a lot of Netflix.”* A primary care nurse practitioner stated, “*I am very religious … ”* In response to the unexpected death of a young patient with COVID-19, she stated, *“I just asked God. ‘Did I say what you needed me to say … did I instill the hope and … the caring that I feel is vital to many of these patients?’”* She stated that her strong beliefs enabled her to see caring for individuals with COVID-19 as “*truly a vocation*”.

Not all experiences were considered negative by participants. Positive relationships were developed with other staff members. As one nurse described, “*While there are very hard days and hard moments, I would say the most meaningful is really recognizing the value and the importance of the role of the nurse.”* One nurse with 23 years of ICU experience noted that what was most meaningful was the camaraderie that developed in the staff, stating, “*when you come to work and take care of these very, very sick patients, you are legitimately putting your life at risk by coming to work, and that really formed sort of a bond between the staff that was there.”*

Going through the experiences together gave participants “*the sense that you’re in it together. You’re just trying to keep everyone alive until seven (end of shift) and … it’s pretty crazy.”* The ability to cope with the vulnerabilities was described as resilience. One nurse participant stated:

*The resilience of the nurses at coming back every day, not knowing what they’re going to be confronted with, dealing with families that are just purely living in fear and the unknown is how they bonded with each other on the unit to help each other out.*

## Discussion

The themes identified in this study were *experiencing vulnerability, suffering loss and grief*, and *coping with vulnerability*. Vulnerability has been identified as the antithesis of resilience (Proag, 2014). The term vulnerability has been used to describe risk associated with disasters, including epidemics and pandemics (Delor & Hubert, 2000). Although the results of a recent study suggested that perceived vulnerability to COVID-19 among frontline nurses during the pandemic did not result in high stress levels (Pasay-an, 2020), the sense of vulnerability apparent in the current study suggested that being vulnerable to a potentially lethal disease along with other disturbing and unexpected experiences was accompanied by high levels of stress among study participants. Angel, Vatne and Martinsen (2020) have suggested that vulnerability is associated with being overwhelmed by one’s feelings and struggling to avoid being harmed. The circumstances encountered by health care workers and first responders in the COVID-19 pandemic—being face-to-face with patients in difficult situations, being unable to relieve patients’ discomfort and anxiety, and witnessing multiple patients’ illness, suffering and death—increased the health care workers’ and first responders’ sense of vulnerability and stress.

The participants in this study described their experience during the COVID-19 pandemic as one that they never expected and one they hoped never to repeat. Their vulnerability arose from their exposure to a virus that resulted in more deaths than World Wars I and II and the Korean and Vietnam wars combined (American Psychological Association, 2020). The acute, deep, and widespread sense of physical and emotional vulnerability experienced by health care workers and first responders has not previously occurred outside of wars and other combat settings. Using language similar to that of nurses who served in Vietnam and the Gulf wars (Rushton, Scott & Callister, 2008; Scannell-Desch, 2005), participants in this study also described their reactions to being on the frontlines and feeling unprepared for what they were facing at the bedside.

Although a number of authors have cautioned about the use and overuse of a war metaphor to describe the COVID-19 pandemic (Bates, 2020; Isaacs & Priesz, 2021; Panzeri, Di Paola & Domaneschi, 2021), officials and others (Biden, 2021; Guterres, 2020; Pasay-an, 2020; Prieto-Merino et al., 2021) continue to refer to the war against COVID-19. Participants in this study described the environments in which they worked as like war zones. A home care nurse described feeling like “cannon fodder,” another reference to wartime with the sense of being considered expendable. Participants’ sense of vulnerability was increased by shortages of PPE and the need by some to provide their own PPE and to justify their requests for a fresh mask from locked PPE supplies. Lack of PPE has been identified across studies as a major factor contributing to health care workers’ and first responders’ sense of vulnerability, risk to themselves for COVID-19 infection, and high stress levels worldwide (Catania et al., 2021; Cohen & van der Meulen Rodgers, 2020; Coşkun Şimşek & Günay, 2021; Fontanini et al., 2021; Galehdar, Toulabi, Kamran & Heydari, 2021; Gordon, Magbee & Yoder, 2021; Joo & Liu, 2021; Shreffler et al., 2020). Participants reported being required to reuse masks designed and intended for single use. Examples shared included having up to 10 exposures to patients with confirmed COVID-19 without appropriate PPE or while reusing PPE after questionable methods of decontamination. Those participants in close contact and interaction with patients reported a high level of exposure to patients with COVID-19. This finding is consistent with other studies in which nurses who were in close contact with patients reported greater exposure to COVID-19 than other health care personnel (Mental Health America, 2021). Study participants have reported feeling abandoned at the frontlines of COVID-19 without PPE, paralleling another reference to the war-like experiences.

The participants’ sense of vulnerability was further increased by inadequate information about the virus responsible for COVID-19, its mode of transmission, and management of the severe respiratory distress and rapid oxygen desaturation that resulted in the death of many patients with COVID-19. Working with staff unfamiliar to them and on patient care units that were also new and unfamiliar to them, along with lack of administrative support, were identified as contributing to their high stress levels. Participants received conflicting information about PPE and patient care protocols that changed quickly. Other researchers (Demirci, Oruc & Kabukcuoglu, 2021) have reported that learning by staff was impeded by the lack of reliable information and resources related to COVID-19.

Study participants’ sense of vulnerability extended to the communities in which they lived. Separation from families by moving away from home or by isolating themselves when in their homes to protect their family members from COVID-19 was a common finding in this and other studies (Coşkun Şimşek & Günay, 2021; De Leo, Cianci, Mastore & Gozzoli, 2021; Fontanini et al., 2021; Gordon et al., 2021; Joo & Liu, 2021; Moradi, Baghaei, Hosseingholipour & Mollazadeh, 2021; Shreffler et al., 2020; White, Wetle, Reddy & Baier, 2021). Study participants felt ostracized by their community and witnessed their children shunned due to their parents’ involvement in caring for individuals with COVID-19. Participants were distressed by the refusal of community members to adhere to requirements for masks and social distancing when workers were risking their lives to care for those with COVID-19. Nurses in other countries have also reported being stigmatized by their community as potential sources of infection (Fontanini et al., 2021; Kackin, Ciydem, Aci & Kutlu, 2020).

Because of administrative decisions, few categories of health care professionals were permitted in the rooms of patients hospitalized with COVID-19. Although staff providing respiratory therapy and obtaining diagnostic X-rays entered patients’ rooms, nurses were expected to shoulder the bulk of responsibilities and activities required to provide hands-on care to patients with COVID-19, a finding also reported by others (Fernández-Castillo, González-Caro, Fernández-García, Porcel-Gálvez & Garnacho-Montero, 2021; Liu et al., 2020; Sun et al., 2020). Nurses felt responsible for meeting the physical and emotional needs of patients that extended well beyond nursing care and interventions. Expectations of staff, particularly nursing staff, were high, and yet administrative support of staff was often viewed as limited. In addition, the training of staff new to these acute and intensive care settings was described as absent or inadequate, increasing participants’ sense of vulnerability and feeling overwhelmed.

Anger and feelings of alienation in addition to frustration towards management and the power inequalities in the workplace have also been reported in other studies (Bennett et al., 2020; Galehdar et al., 2021; Moradi et al., 2021; Yıldırım, Aydoğan & Bulut, 2021). Novice nurses (with less than 6 months of practice) experienced issues similar to those of more experienced health care workers, including fears and concerns, issues with the organization, and the need for support (Garcia-Martin et al., 2021). Loss and grief experienced by study participants during the COVID-19 pandemic related to their sense of loss of control of their lives at work and at home, loss of familiar roles and of support from peers and administrators, separation from supportive family members, and the staggering loss of lives due to COVID-19. Loss of control was exacerbated by administrative decisions to have nurses as the only category of workers to provide all aspects of care as other staff members were prohibited from entering patients’ rooms in an effort to minimize spread of the virus.

In this and other studies, the high mortality rate of patients with COVID-19, particularly in the first wave of the pandemic, produced a profound sense of loss for many participants, who described losing one patient after the other (Ardebili et al., 2021; Gordon et al., 2021). Participants encountered patients who were conversing with them one minute, quickly decompensating and dying soon thereafter, despite all efforts to save their lives. Loss was further exacerbated by the absence of these patients’ families from their bedside due to efforts to contain the virus (Fontanini et al., 2021; Robinson & Stinson, 2021).

Participants’ ability to provide high quality care to their patients was disrupted by the pandemic. The inability to give the quality of care they wanted to provide, not knowing if the treatment protocols were appropriate, questioning if they had done enough to save patients’ lives, and keeping families away from dying patients’ bedside led to strong feelings of guilt and grief among the participants. This was described in other studies as the fear of not measuring up (De Leo et al., 2021), the inability to meet the needs and level of care required by patients with COVID-19 (Danielis et al., 2021), and feeling remorse that the quality of care had decreased (Kackin et al., 2020).

Because of the high expectations of health care workers and first responders during the COVID-19 pandemic, high mortality rates of patients, and perceived lack of support, study participants reported stress, anxiety, depression, inability to sleep, and symptoms associated with PTSD, including flashbacks, panic attacks and nightmares. Others reported fatigue and exhaustion. Similarly, in a study of stress among nurses in Wan, China, Mo et al. (2020) reported that nurses who worked with COVID-19 patients experienced high levels of stress and anxiety. Long working hours and the severity of their patients’ condition contributed to inadequate diets and poor sleep quality, further increasing their stress. In one study (Mental Health America, 2021), a greater percentage of nurses reported high levels of stress and reported receiving inadequate emotional support than other categories of health care workers.

The psychological and emotional impact of COVID-19 reported by participants in this study is similar to that reported by other researchers. Young et al. (2021) reported anxiety and depressive symptoms among health care providers with 5% identifying suicidal ideation and 14% PTSD syndrome. In their scoping review on the impact of COVID-19, Shreffler et al. (2020) reported that health care workers who are women and those who work in high-risk environments—characteristics that describe most of the participants in the present study—may have more negative psychological outcomes than other health care workers. Physical and emotional exhaustion could result in part from the heavy responsibility for patient care borne by nurses, particularly in the absence of adequate support and PPE.

Despite their sense of vulnerability, loss, and grief, the study participants attempted to cope with their experiences. Several described seeking and viewing online information about COVID-19 on their own in response to the lack of information available to them through their work settings. Sharing information from the CDC and other reputable sources with others promoted a sense of collaboration among health care professionals. All participants described decontamination rituals they used to protect their families when they returned home after work.

The need to address the physical and emotional impact of working during the pandemic was discussed by all participants. Their efforts ranged from returning to previous therapy or counselling, engaging in physical exercise, and taking up new or previous hobbies. Although most health care facilities where they were employed generally had available some means of emotional support, information about the programs and support groups was not well disseminated. Thus, few participants reported using them. Support from co-workers was identified as available by most participants. Several found journaling, prayer, and watching movies useful coping strategies. Nurses in Demirci et al.’s (2021) study used similar strategies to cope with their stress levels: physical activity and exercise, waking up and being positive every day, and use of faith-based religious practices or spirituality, yoga, and meditation. Other study participants tried to block out their emotional responses to the events that were occurring around them because they felt that they were unable to correct or control the situation.

It is important to acknowledge the broad consistency of the themes in this study with the quantitative results from the larger CHAMPS study. In the CHAMPS study, 467 hospital-based nurses working during COVID-19 completed four instruments that measured emotional well-being early in the pandemic (Mensinger et al., 2022). Analysis of the survey responses indicated high rates of traumatic stress and depressive symptoms. Future analysis of the CHAMPS study data from a mixed methods perspective has the potential to further explain such consistencies.

## Limitations

A limitation of this study was self-selection, as participants volunteered to be contacted for interviews in their responses in the CHAMPS study. This sample was primarily White non-Hispanic, married, and female. The majority of participants were nurses who worked in inpatient health care facilities in the Northeast region of the United States, although some participants were not nurses and several worked in outpatient settings or in community settings. Although the researchers’ initial goal was to include health care workers from diverse disciplines, relatively few non-nurse health care workers enrolled in the study. Their data are included because they described issues similar to those of nurse participants. Although a number of participants in the CHAMPS study indicated willingness to be contacted for a possible interview, relatively few responded to the requests to schedule interviews. One nurse who declined to be interviewed stated that it would be too distressing to discuss her experiences. Others may have had similar thoughts or did not believe they had further valuable input after their initial participation in the first CHAMPS survey. Alternatively, those who elected not to participate may not have experienced the significant distress and vulnerability reported by study participants. It was noted that few health care professionals who provided direct care to individuals with COVID-19 in long-term care facilities or in home care settings volunteered to participate in the study. Another major limitation of this study is the lack of racial, ethnic, and gender diversity. Although this sample was limited to health care workers and first responders in the U.S., individuals working with patients with COVID-19 throughout the world are at risk for high levels of stress described in this study. Strategies that could have been used to minimize the limitations of this study include more effective recruitment strategies to reach health care workers and first responders from more diverse racial and ethnic populations, health care professionals providing hands-on care for patients in long-term care facilities, and health care workers from more diverse disciplines and settings. Their inclusion is essential to determine if their experiences were similar to those reported in this study.

## Implications of findings

This study has identified a critical need for evidence-based strategies to effectively support health care workers and first responders across disciplines as they cope with prolonged stress, anxiety, fear, and other emotions and as they manage emotional sequelae. Effective strategies are needed to ensure these workers are able to cope with high levels of stress and are adequately prepared to respond to future pandemics as well as be part of an effective workforce. Strategies are needed at the individual level for health care workers and first responders, at the institutional and health care systems levels, at the community or society level, and at national and global levels. Although a number of strategies have been suggested in the literature and through extensive reviews of studies on this topic (Billings, Ching, Gkofa, Greene & Bloomfield, 2021; Halms, Strasser, Kunz & Hasan, 2022; Kovner et al., 2021), the effectiveness of many of these strategies has yet to be determined (Halms et al., 2022).

In their recent scoping review of strategies to support health care workers during the COVID-19 pandemic, Halms et al. (2022) identified four categories of strategies: social/structural support, work environment, communication, and mental health support. Social/structural support strategies should include support from family, friends, partners, and co-workers, as well as efforts to ensure staff retention through providing adequate compensation, rotating staff, and rethinking nonessential work projects. Social support also includes appreciation from employers and from the public, both identified as elusive or non-existent in the current study. Work environment strategies identified by Halms et al. (2022) include a safe and employee-oriented work environment through improved working conditions, the use of best practice protocols, and specific job training and professional development of workers. Other strategies related to the work environment include measures to ensure availability of protective equipment and to minimize risk of transmission of the virus to workers.

Strategies to address communication issues identified by Halms et al. (2022) are essential based on the experiences described by participants in the present study. Communication strategies are needed to ensure reliable and up-to-date information. Lack of clear and consistent communication was repeatedly identified as a factor that increased stress and distress among this study’s participants. Thus, clear communication strategies are essential to ensure that health care workers and first responders are knowledgeable about policies and practices that inform their care of patients and their personal safety.

Halms et al. (2022) also discussed the all-important issue of mental health support of the health care workforce. Of critical importance is the need to identify high-risk individuals showing early signs of mental distress, as well to provide self-help strategies, guidance on resilience, and access to mental health services, either in person or through on-line mechanisms. An important consideration in providing mental health support is the need to do so effectively in a situation in which exhausted workers have little time to spare during their working hours.

In a meta-synthesis of 46 qualitative studies of the COVID-19 and previous pandemics, Billings et al. (2021) identified potential protective strategies for managing the psychological burdens experienced by health care workers. These strategies included increased knowledge about coping strategies, adequate training about procedures and protocols, and formal psychological support. Similar to the report of Halms et al. (2022), Billings et al. described the need for clear, consistent, compassionate messaging; engagement in practical and specific safety and provider training; and institutional supports that provide adequate safety measures including manageable workloads and time away from work. Other protective factors identified by Billings et al. were inclusion of health care workers and first responders in decision-making processes, peer support and emotional guidance from their colleagues.

Kovner et al. (2021) similarly identified the importance of institutions providing resources and services such as consistency and clarity in communication, availability of PPE, emotional support, and promotion of supportive staff relationships and supportive services to minimize the psychological issues that occurred with the COVID-19 pandemic. Of importance is recognition of the need for professional education and development to assist the health care workforce in providing care while simultaneously taking precautions to protect themselves from this potentially lethal disease.

The experiences of loss of lives and resulting grief are important findings of this study. Rabow, Huang, White-hammond and Tucker (2021) reported that grief on the part of health care workers during the COVID-19 pandemic is associated with burnout and distress as members of the health care team experience an unprecedented number of patients deaths, often in very disturbing circumstances and without patients’ family members present. A consequence of the high number of patient deaths, sometimes in rapid succession with little time for reflection and psychological debriefing on the part of health care workers, may be ineffective and unresolved grief. Such grief may be compounded by the sense on the part of health care workers and first responders that their care of patients has been inadequate or not up to usual standards because of staff shortages, uncertainty and changing protocols.

Although the incidence of unresolved or ineffective grief among health care workers during the COVID-19 pandemic is unknown, Rabow et al. (2021) suggests the incidence may be high given the multiple risk factors of health care workers. They further suggest that the mental health burdens associated with the pandemic may last for decades. Thus, strategies are needed for health care workers and first responders to prepare for these deaths, acknowledge these losses, and express grief. Although a number of health care institutions have put into place mental health resources for the health care workforce, these resources see seen as largely invisible by many participants in this study. Strategies to address grief include critical incident stress debriefings, facilitated discussion groups, storytelling, and bereavement counselling (Rabow et al., 2021). These and other strategies need to be tested for effectiveness to ensure an effective workforce now and in future pandemics. Although the COVID-19 pandemic is the first global pandemic since 1918, it is unlikely to be the last. Thus, actions are needed to prevent the devastating effects and events that occurred during the COVID-19 pandemic and threatened the well-being of those who care for patients affected.

## Conclusion

This study adds to the growing body of research on stress among health care workers and first responders during the COVID-19 pandemic and highlights the strong sense of vulnerability experienced by those working with patients with COVID-19 across categories of frontline workers. The highly stressful experiences of health care workers during the pandemic have been compared to wartime experiences by others. Participants’ descriptions of the experiences and their physical and psychological toll suggest that involvement in a battle is not a far-fetched or inappropriate metaphor. Participants identified factors that added to their sense of vulnerability and described efforts and strategies they used to cope with the situation and with the strong emotional reactions they experienced. Although the term vulnerability has been used across disciplines, this concept clearly has applicability to experiences of frontline health care workers and first responders during the COVID-19 pandemic.

Strategies to prevent the negative consequences reported in this and similar studies from occurring in subsequent pandemics have been identified. In addition, strategies to address the sequelae that have affected the physical and mental health of health care workers and first responders in the COVID-19 pandemic have been suggested, although research is needed to determine the effectiveness of these strategies.
